# Two Human Cases of Fatal Meningoencephalitis Associated with Potosi and Lone Star Virus Infections, United States, 2020–2023

**DOI:** 10.3201/eid3102.240831

**Published:** 2025-02

**Authors:** Charles Y. Chiu, Raja Rama Godasi, Holly R. Hughes, Venice Servellita, Kafaya Foresythe, Asritha Tubati, Kelsey Zorn, Sukhman Sidhu, Michael R. Wilson, Sai Varun Bethina, Daniel Abenroth, Yee Cheng, Raymond Grams, Camilla Reese, Carlos Isada, Neeharika Thottempudi

**Affiliations:** Chan–Zuckerberg Biohub, San Francisco, California, USA (C.Y. Chiu); Abbott Pandemic Defense Coalition, Abbott Park, Illinois, USA (C.Y. Chiu, V. Servellita, K. Foresythe); University of California, San Francisco (C.Y. Chiu, V. Servellita, K. Foresythe, A. Tubati, K. Zorn, S. Sidhu, M.R. Wilson); St. Luke’s Boise Medical Center, Boise, Idaho, USA (R. Rama Godasi, D. Abenroth, Y. Cheng, R. Grams, C. Reese); Centers for Disease Control and Prevention, Fort Collins, Colorado, USA (H.R. Hughes); Wake Forest Baptist Medical Center, Winston-Salem, North Carolina, USA (S. Varun Bethina); Cleveland Clinic, Cleveland, Ohio, USA (C. Isada); University of Nevada, Reno, Nevada, USA (N. Thottempudi)

**Keywords:** Potosi virus, Lone Star virus, viruses, bunyavirus, POTV, LSV, arthropod-borne viruses, arboviruses, vector-borne infections, metagenomic next-generation sequencing, mNGS, meningitis/encephalitis, meningoencephalitis, *Amyblomma americanum* tick, *Aedes albopictus* mosquito, United States, ticks, mosquitoes

## Abstract

We used clinical metagenomic next-generation sequencing of cerebrospinal fluid to investigate bunyavirus infections in 2 immunocompromised patients in the United States who had fatal meningoencephalitis. Potosi virus has been isolated from mosquito vectors and Lone Star virus from tick vectors. These findings highlight the power of metagenomic next-generation sequencing in broad-based, agnostic detection of emerging viral infections that test negative using conventional targeted diagnostic methods.

Viruses in the class *Bunyaviricetes* comprise a diverse group of infectious RNA viruses transmitted by arthropods, including mosquitoes and ticks (*1*). Most bunyavirus infections are asymptomatic or manifest as a mild, self-limited febrile illness; however, severe complications, including severe hemorrhagic or neurologic disease, can occur (*2*). We describe fatal bunyavirus infections in 2 immunocompromised patients whose cases we investigated by using clinical metagenomic next-generation sequencing (mNGS) of cerebrospinal fluid (CSF) (*3*,*4*). We also discuss the clinical and public health implications of these findings and the potential utility of mNGS for discovery and surveillance of newly emerging viral pathogens.

## Case 1

In 2020, a 60-year-old man from Ohio, USA, with a history of stage IV non-Hodgkin follicular lymphoma was admitted to the hospital after 4 weeks of progressive cognitive decline, weight loss, headache, and fatigue. He had become bedbound and mute and could not perform activities of daily living. On admission, the patient was noted to be intermittently confused and ataxic with an unsteady gait. Results of a contrast magnetic resonance imaging (MRI) scan of the brain were unremarkable (Figure, panels A, B), showing only moderate diffuse cerebral atrophy with moderately enlarged ventricles consistent with advanced age. Cerebrospinal fluid (CSF) by lumbar puncture on hospital day 2 showed an unremarkable leukocyte count of 1 cell/mm^3^ (reference range 0–5 cells/mm^3^), an elevated erythrocyte count of 67 cells/mm^3^ (reference range 0–5 cells/mm^3^), elevated protein of 75 mg/dL (reference range 15–45 mg/dL), and an unremarkable glucose level of 67 mg/dL (reference range 40–70 mg/dL). Results of a positron emission tomography scan were negative, as were electroencephalogram results, which showed diffuse slowing consistent with encephalopathy and no seizure activity.

**Figure:**
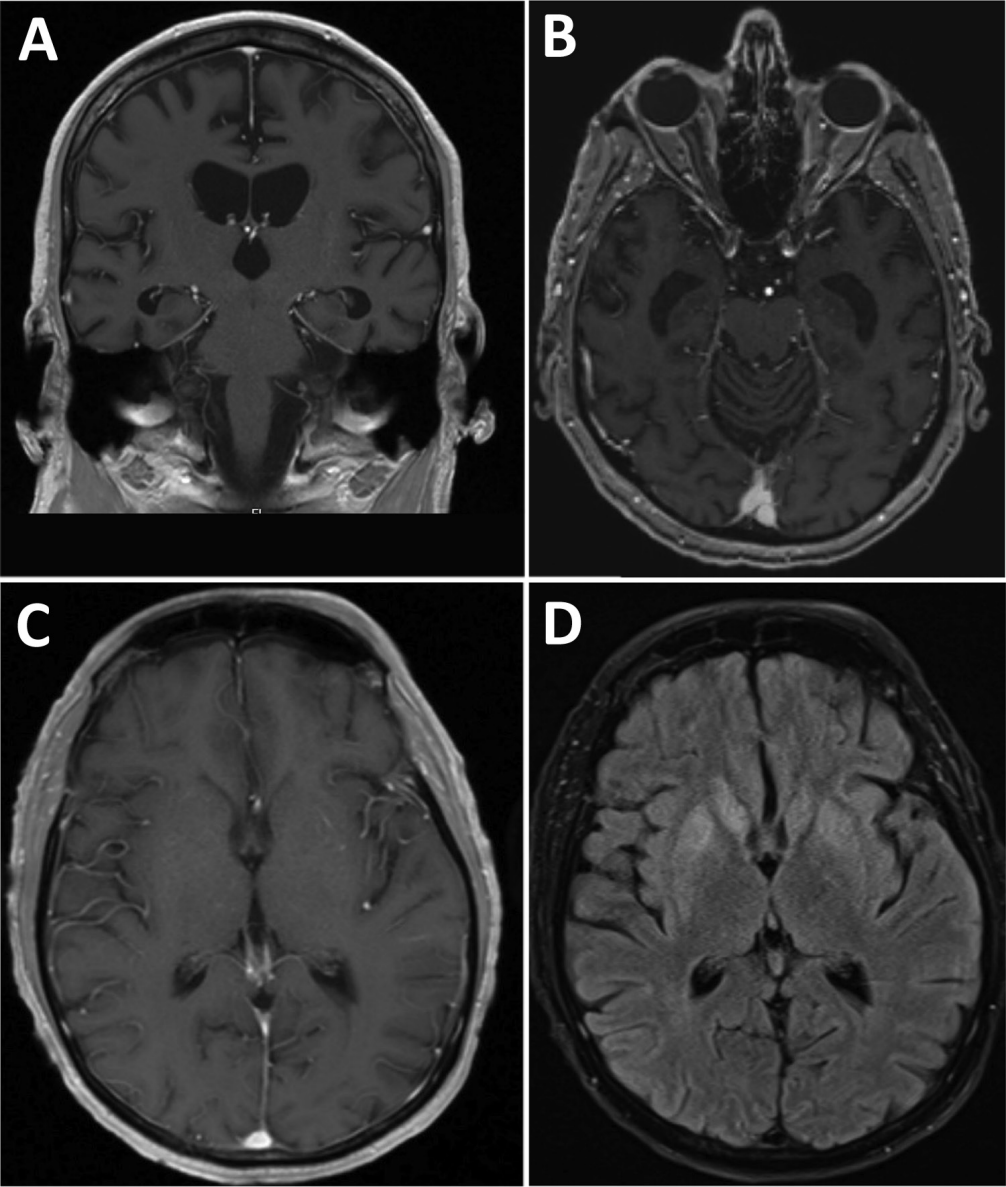
Brain magnetic resonance imaging scans from 2 patients with bunyavirus-associated meningoencephalitis, United States, 2020–2023. A, B) Case-patient 1 brain T1 postcontrast images of coronal (A) and axial (B) sections showing moderately enlarged ventricles and cerebral atrophy. C, D) Case-patient 2 brain T1 postcontrast (C) and T2 postcontrast fluid attenuated inversion recovery (D) images demonstrating bilateral basal ganglia hyperintensities with no contrast enhancement.

The patient resided in rural Ohio and worked at a manufacturing facility. He had completed chemotherapy with rituximab followed by 2 years of maintenance rituximab (completed 4 months before this admission, around the time of symptom onset) and was deemed to be in remission by his oncologist. He was married and monogamous with his wife and had no history of sexually transmitted infection. He reported no international travel in the previous 20 years, as well as no sick contacts, pets, farm animal exposures, or risk factors for tuberculosis.

We administered treatment for possible autoimmune encephalitis with high-dose intravenous methylprednisolone and plasma exchange, which led to minimal improvement. The patient clinically worsened; he had fever, declining mental status, paratonia (increased passive muscle tone), and conjunctivitis with subconjunctival hemorrhages, and we treated him empirically with broad-spectrum antibiotics. CSF examination on hospital day 14 showed unremarkable results for leukocyte count (2 cell/mm^3^), erythrocyte count (0 cells/mm^3^), protein (75 mg/dL), and glucose (57 mg/dL). After transfer to a tertiary care hospital for higher-level care, the patient remained febrile; he had poor mental status and had a new transaminitis of unclear etiology (liver enzyme levels at baseline were within reference ranges). Immunologic evaluation showed severe hypogammaglobulinemia, with total serum IgG levels of 89 mg/dL and CD4 cell counts of 114 cells/mL (reference range 533–1,674 cells/mL). Results of repeat MRI scans of the brain remained negative. We repeated CSF examinations on hospital days 17 and 31, and findings were similar to those previously observed. Extensive autoimmune and microbiologic test results were negative (Table), except for mNGS testing at the University of California San Francisco (UCSF; San Francisco, CA, USA) Clinical Microbiology Laboratory of 2 CSF samples collected on hospital days 4 and 14. We detected Potosi virus (POTV) from both samples. Additional mNGS testing of residual RNA from CSF at the Centers for Disease Control and Prevention (CDC; Fort Collins, CO, USA) detected 87 reads aligning to the small and large segments of POTV. The patient’s neurologic status progressively declined despite supportive therapy, and he died 37 days after admission.

**Table:** Laboratory test results for 2 patients with bunyavirus-associated meningoencephalitis, United States, 2020–2023*

Case-patient and test performed	Sample type	Test site	No. tests	Result
Case-patient 1				
Bacterial, fungal, or AFB culture	CSF, blood	C, T	4	Negative
Mayo autoimmune or neoplastic panel	CSF, serum	Mayo	6	Negative
Cryptococcal antigen	CSF, serum	C	3	Negative
Neurosyphilis test	CSF, serum	C	3	Negative
FilmArray M/E panel	CSF	C	3	Negative
HIV-1 serologic test	Serum	C	1	Negative
Lyme disease serologic test	Serum	C	1	Negative
Hepatitis A–C serologic test	Serum	C	1	Negative
WNV PCR	CSF	T	1	Negative
HSV-1/2 PCR	CSF	T	1	Negative
VZV PCR	CSF	T	1	Negative
HHV-6 PCR	CSF	T	1	Negative
Parvovirus B19 PCR	CSF	T	1	Negative
*Toxoplasma gondii* PCR	CSF	T	1	Negative
*Histoplasma capsulatum* PCR	CSF	T	1	Negative
*H. capsulatum* IgG or IgM	CSF, serum	T	2	Negative
*Coccidioides* IgG or IgM	CSF, serum	T	2	Negative
Adenovirus PCR	CSF	T	1	Negative
(1,3)-𝛽-D glucan assay	CSF, serum	T	2	Negative
Cytologic test	CSF	T	1	Negative
mNGS pathogen diagnosis	CSF	UCSF	2	POTV detected; reads from all 3 segments (S, M, and L)
mNGS	CSF	CDC	1	POTV detected; reads from 2 segments (S and L)
Case-patient 2				
Bacterial, fungal, or AFB culture	CSF, blood	T	3	Negative
Mayo autoimmune/neoplastic panel	CSF, serum	Mayo	1	Elevated GAD65 antibody in serum†
Cryptococcal antigen	CSF, serum	C	3	Negative
*Histoplasma* urinary antigen	Urine	T	1	Negative
Lyme disease serologic test	Serum	T	1	Negative
*Babesia* spp. PCR	CSF	T	1	Negative
*Toxoplasma gondii* PCR	CSF	T	1	Negative
*Histoplasma capsulatum* PCR	CSF	T	1	Negative
*H. capsulatum* IgG or IgM	CSF, serum	T	2	Negative
WNV PCR	CSF	T	1	Negative
HSV-1/2 PCR	CSF	T	1	Negative
FilmArray M/E panel	CSF	T	1	Negative
VZV PCR	CSF	T	1	Negative
HHV-6 PCR	CSF	T	1	Negative
mNGS pathogen diagnosis	CSF	UCSF	2	LSV detected; reads from all 3 segments (S, M, and L)
Virus isolation	CSF	CDC	1	Negative
mNGS	CSF	CDC	1	Negative (no reads to LSV)
PRNT	CSF, serum	CDC	1	Negative

*AFB, acid-fast bacilli; C, community hospital; CDC, Centers for Disease Control and Prevention; HHV-6, human herpesvirus 6; HSV, herpes simplex virus; GAD65, glutamic acid decarboxylase 65; L, large; LSV, Lone star virus; M, medium; M/E, meningitis/encephalitis; Mayo, Mayo Clinic; mNGS, metagenomic next-generation sequencing; POTV, Potosi virus; PRNT, plaque reduction neutralizing testing; S, small; T, tertiary care hospital; UCSF, University of California San Francisco; VZV, varicella zoster virus; WNV, West Nile virus. †0.37 nmol/L (reference range <0.02 nmol/L).

## Case 2

A 60-year-old man from Idaho, USA, with a history of common variable immunodeficiency that was being treated with weekly subcutaneous intravenous IgG was admitted to a tertiary care hospital in 2023 with a 1-week history of headache and myalgias. He had mental status changes, including difficulty with memory, performance of complex motor movements, strange behaviors, and nonsensical conversations, 1 day before hospitalization. He was very active, often hiking and biking outdoors. The patient was retired, owned no pets, and reported no risk factors for tuberculosis or animal or rural exposures.

At admission, the patient was afebrile and alert but confused. Physical examination was unremarkable except for altered sensorium, including the inability to recall his location and various life events. We administered empiric broad-spectrum antibiotics and acyclovir because of a high suspicion of meningoencephalitis. Initial laboratory testing was unremarkable except for mild thrombocytopenia (platelet count of 144,000 [reference range 150,000–450,000]) that reached a nadir of 111,000 on hospital day 4.

CSF obtained on admission showed a lymphocytic pleocytosis with leukocyte count of 6 cells/mm^3^ and 79% lymphocytes, 16% monocytes, and 5% neutrophils; elevated protein of 80 mg/dL, and unremarkable glucose of 66 mg/dL. On hospital day 2, the patient became febrile (temperature up to 38.7°C), so we administered broad-spectrum antibiotics empirically; the patient had unrelenting fevers throughout the rest of the hospital course. On hospital day 3, we performed a repeat lumbar puncture, which continued to show an elevated leukocyte count of 40 cells/mm^3^, elevated protein of 92 mg/dL, and unremarkable glucose of 53 mg/dL. Results of a CSF cytologic test result were negative, as were CSF, serum autoimmune, and serum paraneoplastic panel test results, except for mildly elevated serum glutamic acid decarboxylase 65 antibody of 0.37 nmol/L (reference range <0.02 nmol/L) with corresponding negative CSF glutamic acid decarboxylase 65 antibody.

We performed brain MRI 3 times; the first 2 results were unremarkable, but the third result, obtained on hospital day 14, showed mild interval hyperintensity of the basal ganglia and posterior hippocampi without contrast enhancement (Figure, panels C, D). Continuous electroencephalogram monitoring for 72 hours revealed mild to moderate background slowing with no seizure activity. All microbiologic test results except for CSF mNGS were negative (Table). CSF mNGS testing, which we performed twice on a hospital day 3 sample, was positive for Lone star virus (LSV). Additional testing of residual CSF by CDC did not detect reads from LSV on mNGS, and confirmatory testing with viral isolation and plaque reduction neutralization testing was negative. We attributed the discrepant sequencing results between UCSF and CDC to potential sample degradation caused by multiple freeze–thaw cycles and longer storage times. In addition, differences in sample preparation methods, such as treatment of extracts with DNase (which was performed by UCSF but not by CDC) and kits, could contribute to differences in sensitivity.

The patient remained confused and uncommunicative, and his neurologic status deteriorated such that he could no longer eat or walk. In discussions with his family, we decided to place the patient on comfort measures, and he died 26 days after admission.

## CSF mNGS Analyses

We obtained informed consent from the patients or a surrogate for review of the medical charts under a protocol approved by the UCSF Institutional Review Board (protocol no. 13-12,236). For case 1, the initial sequencing run identified 15 of 4,677,264 reads aligning to Bunyamwera virus in the genus *Orthobunyavirus*, family *Peribunyaviridae* (Appendix Figure, panel A). Of note, the SURPI+ bioinformatics pipeline that was run at the time used a 2016 GenBank reference database (*3*), and the genome of POTV (GenBank accession no. IL94–1899) was deposited in 2017. Thus, POTV was not specifically identified by the SURPI+ pipeline because its genome was not part of the database. By using BLAST (https://blast.ncbi.nlm.nih.gov) to manually align to a 2020 GenBank database and incorporating additional data from a repeat run, we could identify and map 72 POTV reads with coverage of 636 bp (5.1%) of the 12,548-bp trisegmented genome (Appendix Figure, panel A). We identified 50 single-nucleotide polymorphisms (SNPs) within covered regions corresponding to a pairwise identity of 92.1%, and analysis of representative stretches of sequence consisting of <100-bp segments showed that most SNPs were located in the medium segment (Appendix Figure, panel A). We performed multiple sequence alignment of the concatenated genome (with Ns filling the missing regions) and 10 related representative *Orthobunyavirus* genomes by using MAFFT 7.388 (*5*). We conducted maximum-likelihood analysis by using PHYML 3.0 (*6*) to construct a nucleotide phylogenetic tree, which confirmed that the case 1 virus was most closely related to POTV (Appendix Figure, panel A).

For case 2, we identified 50 of 14,386,666 reads aligning to the LSV genome (genus *Bandavirus,* family *Phenuiviridae*) with 84%–95% identity by using the SURPI+ pipeline (Appendix Figure, panel B). Subsequent mNGS runs at high sequencing depth yielded 2,460 reads mapping to all 3 genome segments and covering 1,601 bp (13.6%) of the 11,730-bp trisegmented genome (Appendix Figure, panel B); 183 SNPs corresponded to a pairwise identity of 88.6% within covered regions. Analysis of representative stretches of sequence consisting of <100-bp segments showed that the distribution of SNPs was random but spanned across all 3 segments (Appendix Figure, panel B). We performed multiple sequence alignment of the concatenated genome and 13 related representative genomes in the genus *Bandavirus*. We conducted maximum-likelihood nucleotide phylogenetic analysis by using Gouleako virus as an outgroup, revealing that the case 2 virus was most closely related to LSV (Appendix Figure, panel B). We have submitted partial POTV (n = 5) and LSV sequences (n = 14) to GenBank (accession nos. PQ347819–32).

For both patients, the high nucleotide identity (≈90%) to reference POTV and LSV genomes in GenBank within covered regions and identification of reads from all 3 segments provided confidence in bona fide identification of bunyaviral infections of the central nervous system. Of note, we did not detect bunyavirus reads in the no-template negative control consisting of synthetic CSF matrix in any of the other unrelated patient CSF samples on the sequencing run.

## Conclusions

The class *Bunyaviricetes* comprises a diverse group of >500 arthropodborne or rodentborne viruses characterized by an enveloped trisegmented negative-sense RNA genome (*1*,*7*). Several bunyaviruses, including Rift Valley fever virus, La Crosse virus, Cache Valley virus (CVV), and Jamestown Canyon virus, can cause encephalitis (*2*). We identified 2 bunyaviruses, POTV and LSV, associated with fatal cases of meningoencephalitis in immunocompromised patients. POTV, which is closely related to CVV, was originally isolated from *Aedes albopictus* mosquito pools (*8*); that mosquito species is also an efficient vector for CVV transmission (*9*). LSV, in the genus *Bandavirus*, was isolated from the *Amblyomma americanum* tick (*10*), >10 years after our group reported the genome sequence of LSV (*11*). The key findings of our study include detection of emerging bunyaviruses that had been previously characterized by screening of mosquito and tick populations, underscoring the importance of continued arbovirus surveillance efforts; detection in transplant recipients, who may exhibit atypical or more severe clinical manifestations of infection (*12*); and use of agnostic mNGS for identification of viral infections that are challenging to diagnose (*13*).

Bhanja virus is a bandavirus that was isolated from a *Haemaphysalis intermedia* tick in Bhanjanagar, India, in 1954 and since then has been associated with sporadic cases of febrile illness in encephalitis in humans (*14*). More recently, Heartland virus, another bandavirus, was reported by CDC in 2009 in severely ill patients from the midwestern and southeastern United States with fever, leukopenia, and transaminitis (*15*). Both Heartland virus and LSV use *A. americanum* ticks as a putative tick vector.

The recent emergence of novel bunyaviruses in the United States might be related in part to the increased number of vulnerable persons undergoing transplantation, being treated with immunosuppressive drugs, or both, as well as to changes in the geographic expansion of mosquito and tick vectors that can transmit arboviruses northward and westward (*16*), probably because of climate and environmental factors. An example is a 2023 report of a fatal case of Heartland virus disease acquired in the mid-Atlantic region of the United States (*17*).

This study highlights the importance of broad-spectrum testing such as mNGS for the discovery of emerging viral infections (*13*). Currently, many viral infections are diagnosed by PCR (*18*), which requires the development of targeted primers and probes a priori. In contrast, mNGS is an agnostic approach, by which both known and novel emerging viruses can be identified by their distinct sequence signature (*19*). The minimal CSF pleocytosis in case 1 (1–2 cells/mm^3^) and low to moderate pleocytosis in case 2 (peak leukocyte count of 40 cells/mm^3^) are not necessarily unexpected, given that both patients were severely immunocompromised. In our 7-year longitudinal study of the performance and diagnostic yield of CSF mNGS testing (*20*), we found that the absence of CSF inflammation is observed in more than half of bona fide CNS infections in immunocompromised patients; 39 (53.4%) of 73 UCSF patients exhibited leukocyte counts of <5 cells/mm^3^. A study by Benoit et al. (*21*) also identified several additional CNS bunyavirus infections by metagenomic sequencing, including those caused by CVV (*19*) and Jamestown Canyon virus. Diagnosis of bunyavirus infections typically relies on serologic tests, given that immunocompetent patients rapidly clear the virus and thus results from direct detection testing such as mNGS and reverse transcription PCR are negative. However, serologic testing is problematic for highly immunocompromised patients, for whom results can be false-negative (*22*). In addition, both serologic and molecular testing for several bunyaviruses might only be available in specialized reference laboratories such as at CDC, underscoring the potential utility of mNGS in expanding access to diagnostic testing for patients with bunyaviral encephalitis.

Because mNGS is a molecular detection technique and positive testing alone is insufficient to fulfill Koch’s postulates (*23*), caution is warranted with clinical interpretation of mNGS results, especially with detection of a novel agent of unclear pathogenicity in highly susceptible immunocompromised hosts. Further clinical and epidemiologic studies are needed to characterize the spectrum of clinical disease and pathogenicity associated with POTV and LSV infections in humans. In addition, mNGS is not considered a first-line diagnostic test, given its cost, complexity, and availability only in specialized reference laboratories. The costs of clinical mNGS testing might be reduced once regulatory approval for the test is obtained, prompting more expanded coverage from health insurance providers. Costs might also go down when running the test becomes more efficient, such as by transferring the assay to a commercial partner, thus scaling up access for patients with neurologic infections (*24*). A ≈1-month window occurred for both of our patients during which expedited mNGS testing, with a current laboratory turnaround time of 3–4 days (*19*), might have resulted in an earlier diagnosis. Although no approved treatment for bunyavirus infection is available, the experimental drug favipravir (T-705) is effective against orthobunyaviruses in vitro and in rodent models (*25*–*28*) and efficiently crosses the blood–brain barrier (*29*). Ribavirin also has some activity against bunyaviruses, but its potential efficacy might be limited by poor penetration into the CNS (*30*). Furthermore, we note that in the cases described here, and in many other cases of chronic neurologic bunyavirus infections (*31*), patients specifically have deficiencies in the humoral arm of the immune system. This finding suggests convalescent serum or, in the future, targeted antiviral monoclonal antibodies, could play a role in treatment.

Appendix Metagenomic detection and phylogenetic analysis of Potosi and Lone Star viruses in 2 patients with fatal meningoencephalitis, United States, 2020–2023.
