# Supplementary material for: Two Human Cases of Fatal Meningoencephalitis Associated with Potosi and Lone Star Virus Infections, United States, 2020–2023
Source: Emerg Infect Dis. 2025 Feb;31(2):215–21. doi: 10.3201/eid3102.240831 (PMC11845157; doi:10.3201/eid3102.240831)
Supplement: Appendix — Metagenomic detection and phylogenetic analysis of Potosi and Lone Star viruses in 2 patients with fatal meningoencephalitis, United States, 2020–2023. [file 24-0831-Techapp-s1.pdf]

Article DOI: https://doi.org/10.3201/eid3102.240831

EID cannot ensure accessibility for supplementary materials supplied by authors. Readers who have difficulty accessing supplementary content should contact the authors for assistance.

Two Human Cases of Fatal Meningoencephalitis Associated with Potosi and Lone Star Virus Infections, United States, 2020–2023

Appendix

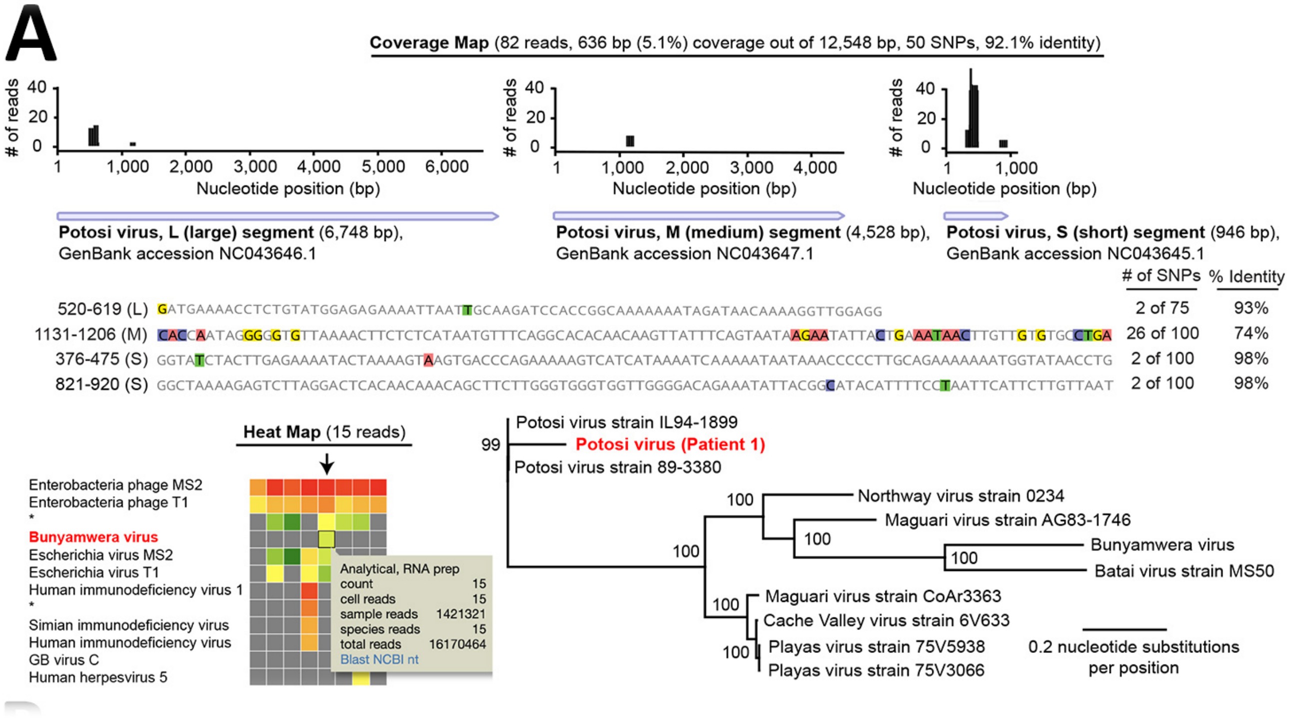

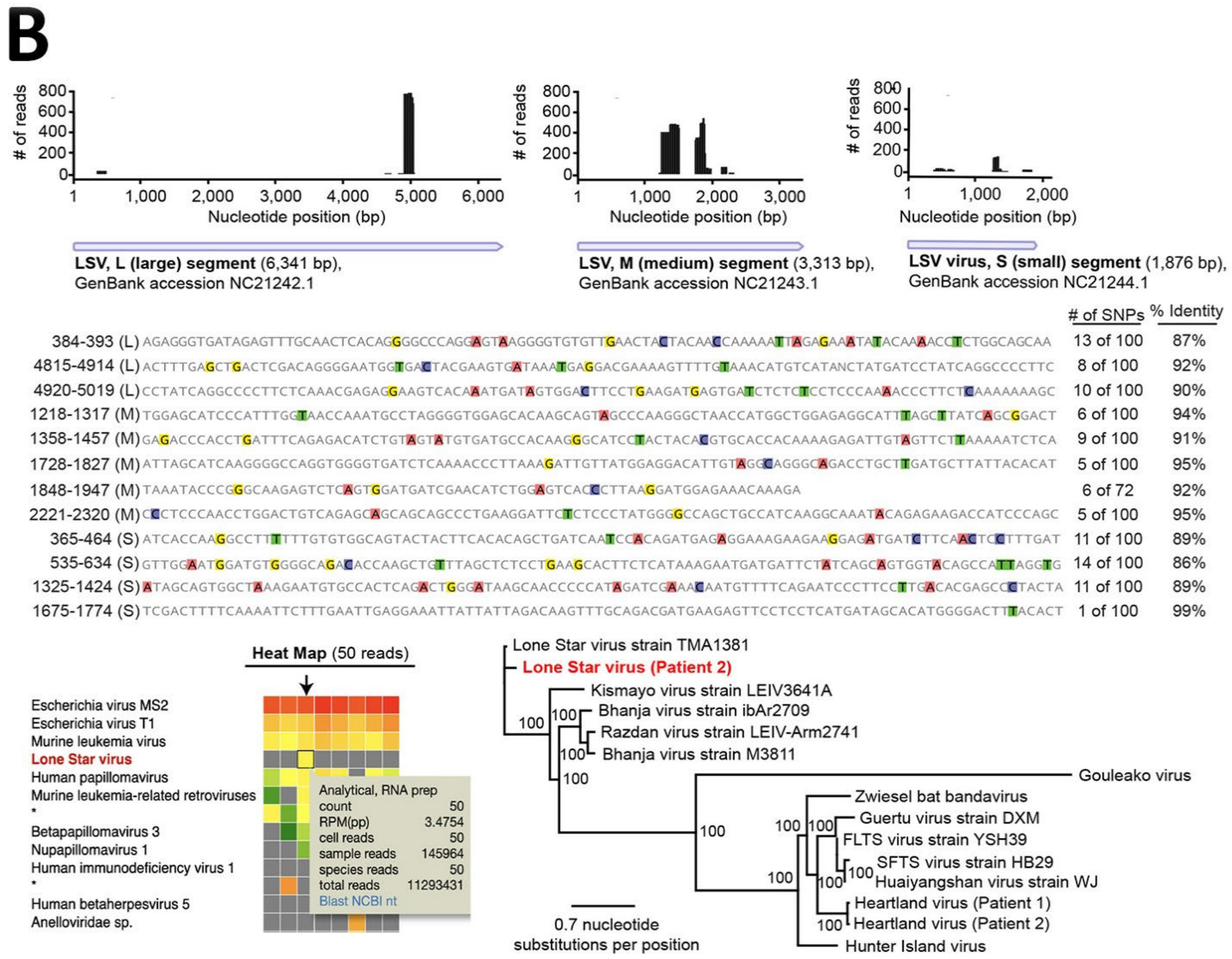

A) Coverage map showing alignment of 72 reads to the 3 segments of the Potosi virus genome (top). Representative sequences of length <100 bp showing distribution of single-nucleotide polymorphisms and pairwise percentage identity compared to the reference Potosi virus genome (middle). Heat map showing detection of reads (n = 15) from an orthobunyavirus with homology to Bunyamwera virus in patient 1 cerebrospinal fluid (bottom left, arrow). Phylogenetic analysis identifies the patient 1 virus as Potosi virus (bottom right). B) Coverage map showing alignment of 2,460 reads to the 3 segments of the Lone Star virus genome (top). Representative sequences of length <100 bp showing distribution of single-nucleotide polymorphisms and pairwise percentage identity compared to the reference Lone Star virus genome (middle). Heat map showing detection of Lone Star virus reads (n = 50) in patient 2 cerebrospinal fluid (bottom left, arrow). Phylogenetic analysis confirms the patient 2 virus to be Lone Star virus (bottom right).
